# Efficacy and Toxicity Profile of Carboplatin/Gemcitabine Chemotherapy in Locally Advanced or Metastatic Biliary Tract Cancer: A Single UK Centre Experience

**DOI:** 10.3390/cancers17193102

**Published:** 2025-09-23

**Authors:** Bahaaeldin Baraka, Dwiti Jatin Ponda, Jennifer Hanna, Dhanny Gomez, Guruprasad Aithal, Arvind Arora

**Affiliations:** 1 Nottingham University Hospitals NHS Trust, Nottingham NG7 2UH, UK; bahaaeldin.baraka@nhs.net (B.B.); dhanny.gomez1@nhs.net (D.G.); 2 University of Nottingham Medical School, Nottingham NG7 2UH, UK; mzydp10@nottingham.ac.uk (D.J.P.); mzyjh27@nottingham.ac.uk (J.H.); 3 NIHR Nottingham Biomedical Research Centre, University of Nottingham, Nottingham NG1 5DU, UK; guru.aithal@nhs.net

**Keywords:** biliary tract cancer, cholangiocarcinoma, chemotherapy, carboplatin, survival outcomes

## Abstract

**Background/Objectives:** Biliary tract cancers (BTCs) refer to an invasive group of malignancies consisting of gallbladder cancer, ampullary cancer, and cholangiocarcinoma (CCA). BTCs are often diagnosed at advanced stages, leading to poor prognoses. While cisplatin-based chemotherapy remains the standard first-line treatment, carboplatin/gemcitabine serves as an alternative, particularly for patients with contraindications to cisplatin. This retrospective study evaluates the real-world efficacy and safety of carboplatin/gemcitabine in patients with advanced BTC who were ineligible for cisplatin-based treatment at a single UK centre. **Methods:** Data from 66 patients treated between March 2018 and July 2023 were analysed for overall survival (OS), progression-free survival (PFS), and toxicities. **Results:** The median OS was 8.97 months (95% CI: 6.78–11.16), and the median PFS was 5.88 months (95% CI: 4.78–6.98). Tumour control rate was achieved in 70% of cases. Patients receiving fewer than four chemotherapy cycles or presenting with poor baseline markers exhibited significantly worse survival outcomes (*p* < 0.05). Haematological toxicities were common, including thrombocytopenia (63.7%), neutropenia (48.5%), and anaemia (50.0%). **Conclusions:** While carboplatin/gemcitabine is a viable treatment for advanced BTC, larger trials are necessary to confirm its efficacy.

## Simple Summary

Biliary tract cancers have a rising incidence and are associated with aggressive, refractory disease and poor prognosis. The current first-line chemotherapy treatment involves cisplatin-based chemotherapy; however, this option is not suitable for patients who are frail, have comorbidities, or are at high risk of nephrotoxicity, neurotoxicity, and ototoxicity. For such patients, carboplatin-based chemotherapy serves as an alternative as it has a more favourable side effect profile than cisplatin. This study is a retrospective analysis that evaluates the use of carboplatin and gemcitabine combination chemotherapy for patients with locally advanced and metastatic biliary tract cancers, by investigating survival outcomes and clinical parameters that influence survival. Of the 66 patients studied, median overall survival was 8.97 months (95% CI: 6.78–11.16), and median progression-free survival was 5.88 months (95% CI: 4.78–6.98). Common toxicities associated with the carboplatin/gemcitabine regimen include thrombocytopenia, neutropenia, and anaemia. This study highlights the feasibility of carboplatin/gemcitabine combination chemotherapy in treating advanced biliary cancers.

## 1. Introduction

Biliary tract cancers (BTCs) are a heterogeneous group of malignancies often associated with poor prognosis and high mortality rates due to delayed presentation. These cancers arise from different parts of the biliary system and can be anatomically classified into gallbladder cancer (GBC) arising from the cystic duct or gallbladder; intrahepatic cholangiocarcinoma (iCCA), which forms in the bile duct inside the liver, i.e., proximal to the second-order bile ducts; extrahepatic cholangiocarcinoma (eCCA), which is found between the site of union of the cystic duct and the common hepatic duct to form the bile duct and the major duodenal papilla; hilar cholangiocarcinoma (HCCA), formerly known as Klatskin tumours, which forms in the biliary tree between second-order bile ducts and the point of insertion of the cystic duct into the common bile duct, including the right and left hepatic duct, and common bile duct up to the point of union with the cystic duct; and ampullary cancer (AC), which is found in the ampulla of Vater, at the site of union between the bile duct and pancreatic duct [1,2,3].

Histologically, more than 90% of BTCs are adenocarcinomas and can be classified as poorly differentiated, moderately differentiated, or well differentiated. Other uncommon histological subtypes include adenosquamous carcinoma, squamous cell carcinoma, mucinous/signet ring cell, lymphoepithelial, clear cell, and undifferentiated variants [4].

In 2021, 216,770 (95% UI: 181,890–245,240) new cases and 171,960 (95% UI: 142,350–194,240) deaths due to BTCs were reported [5]. A significant rise in BTC incidence was observed from 1990 to 2021, with incidence rates increasing by nearly twofold [6]. BTC has been estimated to rank as the sixth most prevalent digestive system cancer worldwide and is the second most common type of primary liver cancer [6,7].

In addition to gallstones and primary sclerosing cholangitis, infections with liver flukes are a major risk factor leading to BTCs being endemic in Southeast Asia and South American countries [8]. This has resulted in considerable global variation in the distribution of BTCs and its disease burden, with notable high-incidence regions including China, India, and Chile [6].

Over half of patients present with treatment-resistant disease [9]. Over a three-decade period, between 1990 and 2017, there was a 76% surge in incidence, a 65% rise in mortality, and a 52% elevation in disability-adjusted life years (DALYs) due to BTCs [10]. Despite being a relatively rare cancer, the mortality rate is substantially marked [11]. The overall survival of patients with gallbladder cancer is less than six months, and for all other types of BTCs, it is under 24 months. The 5-year survival rate is 5–15% [12]. BTCs account for 2% of all cancer-related deaths globally [13].

Based on the results of the ABC-02 trial in 2010, cisplatin/gemcitabine has been established as the standard first-line therapy for locally advanced and metastatic BTCs [14]. As per the ABC-02 trial, cisplatin/gemcitabine doublet therapy has demonstrated superior efficacy compared to gemcitabine monotherapy [14].At present, cisplatin is the cornerstone of first-line therapy for metastatic and locally advanced BTC patients, and based on the findings of the TOPAZ-1 trial, the current standard of care for patients with locally advanced or metastatic biliary tract cancers (BTCs) has evolved to include a combination of cisplatin, gemcitabine, and durvalumab [15]. However, administration of cisplatin is associated with a high risk of nephrotoxicity, neurotoxicity, and ototoxicity [16]. This makes cisplatin unsuitable for certain patient groups, such as those with pre-existing kidney disease, frail and elderly patients, or patients with comorbidities.

Despite these recognised contraindications, the studies conducted to date, ranging from clinical trials to retrospective analyses, have chiefly focused on efficacy and toxicity profiles of cisplatin-based regimens rather than focusing on the subset of patients excluded from treatment due to contraindications. In many of these trials, patients with known contraindications (e.g., severe renal dysfunction) are typically excluded by design, which means that the proportion of the overall biliary tract cancer population that might be ineligible for cisplatin due to contraindications is not explicitly addressed [17,18].

For cases where cisplatin is contraindicated, carboplatin has been proposed as a safer alternative. Carboplatin, a second-generation platinum drug, has a similar mechanism of action to cisplatin and has been proposed as a less toxic and more tolerable alternative for such patients. Furthermore, our dosing strategy aligns with efforts to balance efficacy and safety in vulnerable populations. Compared with standard gemcitabine/carboplatin regimens using AUC 4 or 5 every 3 weeks, our split AUC 2.5 approach prioritises tolerability while maintaining dose intensity. Both carboplatin and cisplatin are alkylating agents, which crosslink genetic material, thereby inhibiting DNA replication. Carboplatin has slower aquation rates compared to cisplatin and is eliminated from plasma at a reduced pace, allowing for a more favourable side effect profile than cisplatin. Carboplatin also exhibits prolonged effects due to lower excretion rates [19]. These factors increase the applicability of carboplatin with the potential to be administered to the high-risk groups mentioned previously. While there is a lack of direct randomised controls in the BTC domain that compare cisplatin and carboplatin, studies have compared cisplatin versus carboplatin for other cancers such as ovarian cancer and urothelial bladder cancer [20,21]. However, data on its real-world efficacy are limited [22].

This study assesses the clinical outcomes and toxicity profiles of patients with advanced BTC receiving carboplatin/gemcitabine at a single UK centre.

## 2. Methods

### 2.1. Study Design

This retrospective observational study was conducted at Nottingham University Hospitals and assessed outcomes in patients diagnosed with locally advanced or metastatic BTC between March 2018 and July 2023 who received carboplatin/gemcitabine chemotherapy. Ethical approval to conduct the audit was obtained through the Clinical Effectiveness Team. Patient confidentiality was maintained in accordance with research governance guidelines.

### 2.2. Patients

Eligible patients were 18 years or older with a histopathological or cytologic diagnosis of BTC, including intrahepatic or extrahepatic cholangiocarcinoma, gallbladder cancer, or ampullary carcinoma. Patients were required to have an Eastern Cooperative Oncology Group (ECOG) performance status of 0, 1, or 2 and an estimated life expectancy exceeding three months. Patients who had received any form of previous therapy, such as surgery or chemotherapy, were not excluded from this study. Additional eligibility criteria included adequate haematological and biochemical functions, defined as bilirubin ≤ 1.5 times the upper limit of normal, liver enzyme levels ≤ five times the upper limit of normal, and carboplatin dosing calculated based on glomerular filtration rate (GFR), with administration permitted only in patients with a GFR ≥ 20 mL/min (Table S1, Supplementary Materials). Power calculations were not necessary due to the retrospective study design and restricted availability of data, leading to a pre-defined sample size.

### 2.3. Treatment

Patients received carboplatin based on the area under the curve (AUC), with a dose of AUC 2.5 on days 1 and 8 of a 21-day cycle, to better accommodate patient frailty while reducing side effects and improving tolerability of the treatment. This was combined with gemcitabine (1000 mg/m^2^) on days 1 and 8, for a duration of up to 24 weeks, with interim radiological response assessments every 3 months (12 weeks) to monitor progress. This modified dosing schedule was selected based on previous research supporting split-dose carboplatin to enhance tolerability in frail and elderly [23]. Dose modifications were permitted based on haematological toxicity, renal function abnormalities, and adverse events such as nausea, vomiting, peripheral neuropathy, or lower limb oedema, in accordance with the SmPC criteria for dose modification. Treatment was discontinued upon disease progression, unacceptable toxicity, or at the discretion of the patient or clinician.

### 2.4. Assessments

Clinical assessments were conducted at the start of each chemotherapy cycle, including a physical examination, laboratory tests, and toxicity evaluations. Tumour response was measured according to Response Evaluation Criteria in Solid Tumours (RECIST) 1.1, with imaging assessments conducted at week 12 and again at week 24 in patients completing treatment. Tumour control was defined as complete response, partial response, or stable disease. Disease progression was based on radiological criteria or the emergence of new metastatic disease. Patients were followed every three months after treatment for survival assessment, and late toxicity evaluation and support.

### 2.5. Data Extraction and Statistical Analysis

Data extraction using a secured Excel sheet was carried out between 10 October 2023 and 22 November 2023. Data were extracted based on patient characteristics and demographic information, clinical parameters, disease characteristics, outcome measures, treatment details, and toxicity profiles. Table S1 in the Supplementary Materials contains details about data extraction parameters.

Due to a lack of sufficient data for patients’ full molecular profiling, grade of tumour, and TMN staging, the parameters were removed before data analysis was conducted.

The primary outcome of this study was to examine the overall survival (OS), progression-free survival (PFS), and toxicities, as well as tumour response. OS was measured in months between the date of the first cycle of chemotherapy and the date of death. PFS was calculated in months between the date of the first cycle of chemotherapy and the date of confirmed radiological progression. In the case of patient death before the date of radiological progression, the date of death was used to calculate PFS.

Disease response was identified from radiological records in accordance with the Response Evaluation Criteria in Solid Tumours (RECIST). Complete Response is the disappearance of all target lesions. Partial Response is characterised as a minimum of 30% tumour shrinkage. Progressive disease is classified as more than 20% growth in tumour size. Stable disease is when the tumour has not grown beyond 20% or shrunk by more than 30%. The tumour control rate (TCR) was calculated as the percentage of patients who had a complete or partial response or stable disease from baseline.

Furthermore, survival outcomes were analysed using Kaplan–Meier curves and log-rank tests. Cox proportional hazard models estimated hazard ratios and assessed prognostic factors. Toxicities were classified using the National Cancer Institute’s Common Toxicity Criteria for Adverse Events (CTCAE), version 4.0. All statistical analyses were conducted using SPSS Statistics for Windows, Version 28.0.1.

## 3. Results

### 3.1. Baseline Patient Characteristics

Data from a total of 66 patients were eligible for analysis. The median age of the patients analysed was 72 years. The median follow-up time for the patients was 7.5 months, calculated using the reverse Kaplan–Meier method, which accounts for censored observations. The median number of cycles of carboplatin/gemcitabine chemotherapy administered to patients was 4 cycles.

In the patient population, 9.1% of patients had gallbladder cancer, 53% of patients had intrahepatic cholangiocarcinoma, and 22.7% of patients had extrahepatic cholangiocarcinoma. The remaining 9.1% and 6.1% had hilar cholangiocarcinoma and ampullary cancer, respectively. Furthermore, at the point of diagnosis, 65.2% of patients had metastatic disease, and 34.8% of patients had locally advanced disease. Patient characteristics were analysed as shown in Table S2 (Supplementary Materials).

Upon analysis of baseline patient factors that contradicted cisplatin administration, the most prevalent reason was pre-existing kidney disease in 32.65% of patients. Other frequently observed reasons for contraindication include comorbidities like metabolic and vascular disease (26.53%) and old age and frailty (22.45%).

### 3.2. Outcome: Overall Survival and Progression-Free Survival

#### Overall Survival and Progression-Free Survival

**Figure 1:**
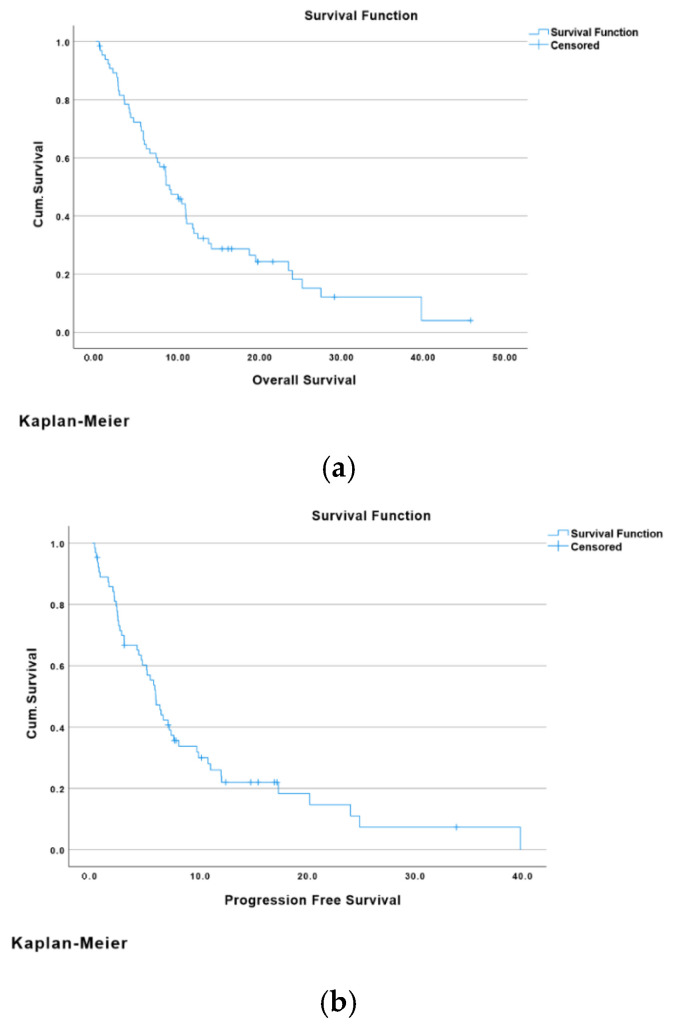
(**a**) The median overall survival (OS) for all types of biliary tract cancers; (**b**) The median progression-free survival (PFS) for all types of biliary tract cancers.

A Kaplan–Meier curve was used to analyse survival over time. The median overall survival (OS) for all types of biliary tract cancers was 8.97 months (95% CI: 6.78–11.16), as shown in Figure 1a. However, OS varied based on the primary site of biliary tract cancer. The median OS for gall bladder cancer was 6.57 months (95% CI: 0.85–12.29). For intrahepatic and extrahepatic cholangiocarcinoma, the median OS was 7.46 months (95% CI: 3.73–11.19) and 10.94 months (95% CI: 0–23.13), respectively. Patients with hilar cholangiocarcinoma had a median OS of 9.17 months (95% CI: 1.63–16.71), whereas those with ampullary cholangiocarcinoma had a median OS of 14.1 months (95% CI: 5.28–22.92).

On the other hand, the median progression-free survival (PFS) for all types of biliary tract cancers was 5.88 months (95% CI: 4.78–6.98), as shown in Figure 1b. This, too, differed based on the primary location of the cancer. For gall bladder cancer, the median PFS was 2.96 months (95% CI: 0–8.01). The median PFS for intrahepatic and extrahepatic CCA was 5.78 months (95% CI: 3.57–7.99) and 6.37 months (95% CI: 3.82–13.86), respectively. Patients with hilar cholangiocarcinoma had a median PFS of 5.88 months (95% CI: 4.76–6.99), whereas those with ampullary cholangiocarcinoma had a median PFS of 8.02 months (95% CI: 1.87–14.17).

The Kaplan–Meier curve for subgroup analysis of OS and PFS for different types of BTCs is provided in the Supplementary Materials (Table S3). Log-rank tests were performed to compare the differences in survival among subgroups, as shown in Table S3 (Supplementary Materials).

### 3.3. Tumour Response and Survival Status

#### Disease Control Rate

At the 3-month evaluation, disease response data were available for 40 patients. Among them, 26 patients (65%) exhibited stable disease, 2 patients (5%) showed a partial response, and 12 patients (30%) had progressive disease. Accordingly, the disease control rate (DCR), defined as the proportion of patients with stable disease or partial response, was 70%.

At the 6-month follow-up, disease response data were available for 38 patients. Of these, 14 patients (36.8%) had stable disease, 3 patients (7.9%) showed a partial response, and 21 patients (55.3%) had progressive disease. Thus, the disease control rate (DCR) at 6 months was 44.7%.

The 6-month survival rate was 62.1%, while the 12-month survival rate declined to 31.8%. Table S6 in the Supplementary Materials demonstrates the distribution of survival outcomes (Alive vs. Dead) over time points (6 Months, 12 Months, End of Study) in the study cohort.

Both OS and PFS improved when patients received more than four cycles of carboplatin gemcitabine therapy (*p* < 0.05). In contrast, baseline CA 19-9 values greater than 2000 U/mL and baseline alkaline phosphatase values higher than 180 IU/L were associated with a significant (*p* < 0.05) decrease in OS and PFS.

Further statistical analyses examining the association of patient characteristics with OS and PFS, including multivariate analysis (Tables S4 and S5) and bivariate analysis (Tables S7 and S8) are presented in the Supplementary Materials.

### 3.4. Toxicity Profiles

Toxicities from chemotherapy were systematically analysed. Grade 3 and 4 haematological toxicities were reported in 51.5% of patients. Thrombocytopenia was the most frequently observed adverse event, affecting 63.7% of patients. Additionally, anaemia and neutropenia were reported in over 50% of cases. Non-haematological toxicities reported include nausea and vomiting in 31.8% (*n* = 21) of patients, fatigue in 53.03% (*n* = 35) of patients, and peripheral neuropathy in 15.2% (*n* = 10) of patients. Gastrointestinal side effects, including constipation, diarrhoea, and dyspepsia, were reported in 22.7% (*n* = 15) of patients. Other uncommon side effects such as epistaxis were reported in 4.54% (*n* = 3) patients, and oedema was reported in 1.52% (*n* = 1) of patients. A detailed breakdown of adverse events by grade (G1–2 vs. G3–4) is provided in Table S9 (Supplementary Materials). Febrile neutropenia occurred in 3.0% (*n* = 2) of patients, while 12.1% (*n* = 8) of patients required blood transfusions. Moreover, 13.6% (*n* = 9) of patients received G-CSF support, and 4.5% of patients (*n* = 3) required hospitalisation for treatment-related complications. Notably, there were no deaths or intensive care admissions directly attributable to treatment, despite the high rates of grade 3–4 haematological toxicities. No treatment-related deaths or irreversible toxicities were observed.

## 4. Discussion

Careful consideration of contraindications to cisplatin is important to ensure patient safety and prevent adverse events. Our findings indicate that carboplatin/gemcitabine provides a viable treatment alternative for patients who are ineligible for cisplatin-based standard regimens. For such patients, administering carboplatin instead of cisplatin can increase survival while maintaining quality of life.

When compared to the results of Valle et al.’s 2010 ABC-02 study [14], which evaluated the efficacy of cisplatin/gemcitabine, the OS and PFS reported in our study are inferior. The OS and PFS for the cisplatin/gemcitabine are 11.7 months and 8.1 months, respectively. However, direct comparison is limited by fundamental methodological differences such as study design, baseline population characteristics, and sample size. The ABC-02 study had stringent inclusion and exclusion criteria. For instance, patients with prior chemotherapy were not included in the ABC-02 study, whereas in our study 30.3% received previous chemotherapy, and 63.67% of patients received treatment prior to carboplatin/gemcitabine administration. Additionally, the regimen demonstrated efficacy in subpopulations, particularly ampullary and extrahepatic CCA. Ethnic and regional variations in treatment response further complicate the universal applicability of the ABC-02 findings. For instance, studies in specific populations, such as those from Korea, have reported that the benefits of gemcitabine combined with cisplatin over gemcitabine monotherapy are not as apparent, likely due to differences in genetic, environmental, and practice-related factors that were not adequately controlled in the ABC-02 trial [24].

Pre-existing research evaluates several alternative chemotherapy regimens for BTCs. The results from a phase II randomised trial evaluating sorafenib for BTC demonstrated an OS of 9 months and PFS of 3 months [25]. Furthermore, the findings from a 2006 study by Okusaka et al. [26] demonstrated that treatment with gemcitabine monotherapy was associated with a tumour control rate (TCR) of 55%, a median overall survival (OS) of 7.6 months, and a median progression-free survival (PFS) of 2.6 months [26]. Similarly, the analysis of oxaliplatin/gemcitabine dual therapy exhibited a TCR, OS, and PFS of 71%, 11 months, and 6.5 months, respectively [27].The results of our study have shown comparable efficacy measured in OS, PFS, and TCR. Nevertheless, cross-trial comparisons should be interpreted with caution, given the differences in study methodologies.

To our knowledge, there is only one study that has analysed the carboplatin/gemcitabine regimen in BTCs, namely the one by Williams et al. in 2010 [28]. The prospective study found a PFS of 7.8 months (versus 5.88 months in the current study) and OS of 10.6 months (versus 8.97 months in the current study). This can be potentially attributable to differences in baseline patient characteristics and study design. Notably, patients in our study had a higher median age (72 vs. 63 years) and were more likely to have metastatic disease at diagnosis.

For the carboplatin/gemcitabine regimen analysed here, the toxicity profile was notable for haematological complications, aligning with previous studies.

The shortcomings of this study include a limited sample size, which may not represent a wider population, introducing low population validity. This is also a single-centre study and may not reflect practices in most institutions, overlooking variations in practice across the country. Thus, this study is likely to have low external validity. These factors may also impact the generalisability of the study. Additionally, the retrospective study design is constrained in establishing causation and does not effectively balance confounding variables. There are also temporal limitations, and some long-term trends may have been missed in the analysis. A key limitation of our analysis is the inability to stratify survival outcomes by line of therapy, ECOG performance status, or extent of disease (metastatic vs. locally advanced), due to dataset constraints. While these factors undoubtedly impact OS and PFS, our current cohort size and data completeness did not allow for meaningful subgroup analysis.

Nevertheless, carboplatin/gemcitabine has shown a promising role in managing BTCs, and our study strives to inform evidence-based practices in this research domain. Despite these limitations, our study underscores the need for further research, including prospective trials, to validate carboplatin’s role in BTC management [29]. It is important to note that there is a paucity of head-to-head, direct, randomised controlled trials to guide clinical practice in this domain. Moreover, differences in population characteristics, variation in endpoints, and lack of rigorous stratification have hindered progress in the field [30].

## 5. Conclusions

Carboplatin/gemcitabine presents a feasible treatment option for advanced BTC patients ineligible for cisplatin. While survival outcomes are modest compared to cisplatin, this regimen offers a well-tolerated alternative with manageable toxicity, which can have a significant positive impact on quality of life. These findings have the potential to guide clinical practice. Larger-scale prospective studies are warranted to refine treatment strategies and improve patient outcomes.

## Data Availability

Data are contained within the article and Supplementary Materials.

## Supplementary Materials

The following supporting information can be downloaded at https://www.mdpi.com/article/10.3390/cancers17193102/s1, Table S1: Parameters used in data extraction; Table S2: Baseline Patient Characteristics; Table S3: PFS log rank test for PFS and OS; Table S4: Multivariate analysis of Overall Survival; Table S5: Multivariate analysis of PFS; Table S6: Bivariate analysis of Survival status; Table S7: Bivariate analysis of Survival status at 6 months; Table S8: Bivariate analysis of survival status at 12 months; Table S9: Frequency table of toxicities from chemotherapy.
