# Supplementary material for: Efficacy and Toxicity Profile of Carboplatin/Gemcitabine Chemotherapy in Locally Advanced or Metastatic Biliary Tract Cancer: A Single UK Centre Experience
Source: Cancers (Basel). 2025 Sep 23;17(19):3102. doi: 10.3390/cancers17193102 (PMC12523769; doi:10.3390/cancers17193102)
Supplement: Supplementary file 1 [file cancers-17-03102-s001.zip › cancers-3854819-supplementary.pdf]

Supplementary Materials

S1. Data Extraction

Parameters included demographic information, disease characteristics, clinical parameters, outcome measures and treatment details [Table S1].

Table S1. Parameters used in data extraction.

Demographic Information	Disease Characteristic	Clinical Parameters	Treatment Details	Outcome Measures
Age ≥18 years old	Extent of Disease	ECOG Performance Score	No. of cycles	Overall Survival
Sex	Primary Tumor Site and Type of Tumor	Reason for not giving cisplatin	Dosage reductions and reason	Progression Free Survival
	Tumor size, grade, lymph node involvement and metastasis sites	Type of Previous therapy (if any)	Follow up period	Toxicity Profile and Grade of Toxicities
	Genomic and Molecular Markers- FGFR status	Pre-chemotherapy bilirubin, albumin, and Alkaline Phosphate ALP values	Second-line therapy (if any)	Treatment Response- using Radiological Data (Disease Progression, Stable Disease or Partial Response)
		Pre-chemotherapy Hemoglobin, Neutrophile and Lymphocyte Count		

S2. RESULTS

S2.1 Baseline Patient Characteristics [Table S2]

Table S2. Baseline Patient Characteristics.

Characteristics	Frequency n (%)
**Sex**	
Male	39 (59.1)
Female	27 (40.9)
**ECOG Performance Score**	
0	5 (7.6)
1	51 (77.3)
2	10 (15.2)
**Extent of Disease**	
Locally Advanced	23 (34.8)
Metastatic	43 (65.2)
**Site Status**	
Gall Bladder	6 (9.1)
Intra-Hepatic CCA	35 (53)
Extra-Hepatic CCA	15 (22.7)
Hilar	6 (9.1)
Ampullary	4 (6.1)
**Previous Therapy Status**	
Yes	24 (36.4)
No	41 (62.1)
Missing Data	1 (1.5)
**Type of Previous Therapy**	
Curative or Palliative Surgery	9 (13.64)
Biliary Stent	18 (27.27)
Chemotherapy	20 (30.3)
Others including Radiotherapy	1 (0.015)

**Previous Chemotherapy**		
No	50 (75.8)	
Yes	16 (24.2)	
**Baseline CA19.9 Values**		
CA19.9<2000	52 (78.8)	
CA19.9>2000	14 (21.2)	
**Baseline Haemoglobin (Hb) Values**		
Hb<120	28 (42.4)	
Hb>120	38 (57.6)	
**Baseline Albumin (ALB) Values**		
ALB<34	25 (37.9)	
ALB>34	39 (59.1)	
**Baseline Bilirubin Values**		
Bilirubin<13	34 (51.5)	
Bilirubin>13	25 (37.9)	
**Baseline Alkaline Phosphate (ALP) Values**		
ALP<180	32 (48.5)	
ALP>180	31 (47)	
**Neutrophil-Lymphocyte Ratio (NLR)**		
NLR<5	47 (70.1)	
NLR>5	12 (17.9)	

S2.2 log rank test for PFS and OS [Table S3]

Table S3. PFS log rank test for PFS and OS.

Variable	Overall Survival (OS)				Progression Free Survival (PFS)			
	Median OS	95% CI		p value	Median PFS	95% CI		p value
		Lower	Upper			Lower	Upper	
Pre-Chemo CA19.9 Value	8.97	8.632	13.248		5.88	4.779	6.981	
CA 19.9<127	10.94	7.189	9.771	0.527	6.57	4.56	8.58	0.305
CA19.9>127	8.48	6.778	11.162		5.03	1.913	8.147	
LN involvement Status	8.97	6.778	11.162		5.88	4.779	6.981	
No LN involved	11.8	6.882	16.718	**0.019**	5.91	3.703	8.117	0.1
LN involved	6.57	3.62	9.52		5.78	3.465	8.095	
Extent of Disease	8.97	6.778	11.162		5.88	4.779	6.981	
Locally Advanced	13.73	4.189	23.271	0.141	7.03	1.256	12.804	0.358
Metastatic	8.54	6.596	10.484		5.78	2.088	7.981	
ECOG Performance Score	8.97	6.778	11.162		5.88	4.779	6.981	
0	11.8	7.978	15.622	0.48	8.02	0	16.448	0.093
1	8.54	5.825	11.255		5.88	4.518	7.242	
2	5.42	0.369	10.471		2.942	0	6.459	

Previous Chemotherapy Status	8.97	6.778	11.162		5.88	4.779	6.981	
No previous chemotherapy	8.97	6.794	11.146	0.952	5.91	4.781	7.039	0.778
received previous chemotherapy	7.46	0.58	14.34		5.68	2.91	8.45	
Baseline Haemoglobin Values	8.97	6.778	11.162		5.88	4.779	6.981	
Hb<120	7.46	4.31	10.61	0.489				
Hb>120	10.02	7.327	12.713					
Baseline Bilirubin Values	8.97	6.778	11.162		5.88	4.814	6.946	
Bili<13	11.8	9.833	13.767	**<0.01**	7.3	4.565	10.035	0.35
Bili>13	5.78	3.618	7.942		5.06	1.388	8.732	
Baseline Albumin Values	5.78	6.601	11.739		5.85	4.446	7.254	
Alb<34	5.78	5.144	6.416	**0.012**	4.3	1.715	6.885	**0.007**
Alb>34	10.9	8.327	13.473		7.3	4.882	9.718	
Baseline Alkaline Phosphate Values	8.54	6.101	10.979		5.85	4.522	7.178	
ALP<180	11.96	7.843	16.077	**<0.001**	9.86	2.349	17.371	**<0.001**
ALP>180	5.88	3.797	7.963		4.63	1.311	7.949	
Neutrocyte-Lymphocyte Ratio	8.54	6.793	10.287		5.85	4.545	7.155	
NLR<4	11.96	7.669	16.251	**<0.001**	6.57	4.603	8.537	**0.009**
NLR>4	6.11	3.126	9.094		4.53	1.505	7.555	

Number of Cycles Received	8.54	6.082	10.998	**<0.001**	5.85	4.542	7.158	**0.005**
<4	5.42	3.458	7.382		2.942	0.484	5.4	
>4	13.73	9.824	17.636		8.02	4.696	11.344	

S2.3 Multivariate analysis to determine association of patient characteristics with OS and PFS as depicted in [Table S4] and [Table S5] respectively.

Table S4. Multivariate analysis of Overall Survival.

Variable (Code)	B	P value	Hazard Ratio- Exp (B)	95% CI for Exp (B)	
				Lower	Upper
Sex<br>Male (1)<br>Female (2)	-1.092	0.016	0.336	0.138	0.813
Age<br>Age<72 (0)<br>Age>72 (1)	-0.049	0.904	0.952	0.429	2.116
ECOG PS<br>(0,1,2)	-0.772	0.191	0.462	0.145	1.471
Number of Cycles<br>No. of Cycles<4<br>No. of Cycles>4	-1.814	**0.001**	0.163	0.054	0.49
Extent of Disease<br>Locally Advanced (0)<br>Metastatic (1)	10.207	0.878	27087.35	0	1.084 E+61
Site Status<br>GB (1)<br>IH-CCA(2)<br>EH- CCA (3)	-0.121	0.634	0.866	0.538	1.459

Hilar (4)<br>Ampullary (5)					
Previous Chemotherapy Status<br>No previous chemotherapy (0)<br>Previous Chemotherapy (1)	-0.684	0.259	0.505	0.154	1.655
Lymph node Involvement<br>No LN involvement (0)<br>LN involvement (1)	0.344	0.414	1.411	0.618	3.221
Baseline Bilirubin Values<br>Bili<13 (0)<br>Bili>13 (1)	0.349	0.512	1.417	0.5	4.012
Baseline Albumin Values<br>Alb<34 (0)<br>Alb>34 (1)	-0.622	0.278	0.537	0.174	1.654
Baseline Alkaline Phosphate Values<br>ALP<180 (0)<br>ALP>180 (1)	0.12	**0.001**	0.163	0.364	3.499
Neutrophil to Lymphocyte Ratio<br>NLR<4 (0)<br>NLR>4 (1)	0.799	0.088	2.223	0.889	5.557
Baseline Haemoglobin Value<br>Hb<12<br>Hb>12	-0.234	0.624	0.791	0.311	2.016
Pre-chemo CA19.9<br><2000<br>>127	1.274	**0.004**	3.575	1.486	8.603

Dose Reduction Status<br>No Dose Reduction (0)<br>Dose Reduction (1)	0.315	0.521	1.37	0.524	3.583

Table S5. Multivariate analysis of PFS.

Variable (Code)	B	P value	Hazard Ratio-Exp (B)	95% CI for Exp (B)	
				Lower	Upper
Sex<br>Male (1)<br>Female (2)	-0.739	0.104	0.478	0.196	1.164
Age<br>Age<72 (0)<br>Age>72 (1)	0.368	0.38	1.444	0.636	3.281
ECOG PS<br>(0,1,2)	-0.064	0.906	0.938	0.324	2.715
Number of Cycles<br>No. of Cycles<4<br>No. of Cycles>4	-1.843	**0.002**	0.158	0.049	0.509
Extent of Disease<br>Locally Advanced (0)<br>Metastatic (1)	11.125	0.887	67871.71	0	4.70E+71
Site Status<br>GB (1)<br>IH-CCA(2)<br>EH- CCA (3)<br>Hilar (4)<br>Ampullary (5)	-0.107	0.707	0.899	0.514	1.57
Previous Chemotherapy Status<br>No previous chemotherapy (0)	-0.57	0.371	0.566	0.162	1.972

Previous Chemotherapy (1)					
Lymph node Involvement<br>No LN involvement (0)<br>LN involvement (1)	0.328	0.437	1.389	0.607	3.179
Baseline Bilirubin Values<br>Bili<13 (0)<br>Bili>13 (1)	-1.276	**0.03**	0.279	0.088	0.886
Baseline Albumin Values<br>Alb<34 (0)<br>Alb>34 (1)	-0.663	0.236	0.515	0.172	1.544
Baseline Alkaline Phosphate Values<br>ALP<180 (0)<br>ALP>180 (1)	0.681	**0.002**	0.163	0.676	5.769
Neutrophil to Lymphocyte Ratio<br>NLR<4 (0)<br>NLR>4 (1)	0.332	0.487	1.393	0.547	3.545
Baseline Haemoglobin Value<br>Hb<12<br>Hb>12	0.272	0,586	1.312	0.493	3.49
Pre-chemo CA19.9	1.329	**0.003**	3.779	1.566	9.118
Dose Reduction Status<br>No Dose Reduction (0)<br>Dose Reduction (1)	-0.03	0.953	0.97	0.357	2.638

S3. Survival Status

S3.1 Bivariate analysis of Survival status [Table S6]

Table S6. Bivariate analysis of Survival status.

CRITERIA	SURVIVAL STATUS		Pearson Chi Square		
	ALIVE	DECEASED	Value	df	χ^2 p-value
Sex					
Male	8 (20.5)	31 (79.5)	0.04	1	0.841
Female	5 (18.5)	22 (81.5)			
Age					
Age <72	7 (19.4)	29 (80.6)	0.03	1	0.955
Age>72	6 (20)	24 (80)			
Extent of Disease					
Locally Advanced	9 (39.1)	14 (60.9)	8.429	1	**0.04**
Metastatic	4 (9.3)	39 (90.7)			
Primary Cancer Site					
Gall Bladder	0	6 (100)	2.04	4	0.728
Intra-Hepatic CCA	7 (20)	28 (80)			
Extra-Hepatic CCA	4 (26.7)	11 (73.3)			
Hilar	1 (16.7)	5 (83.3)			
Ampullary	1 (25)	3 (75)			
ECOG PS					
0	1 (20)	4 (80)	2.927	2	0.231
1	12 (23.5)	39 (76.5)			
2	0	10 (100)			
Lymph node involvement					
No LN involvement	8 (22.2)	28 (77.8)	0.319	1	0.572
LN involvement	5 (16.7)	25 (83.3)			
Previous Therapy Status					
No Previous Therapy	7 (29.2)	17 (70.8)	1.998	1	0.157

Received Previous Therapy	6 (14.6%)	35 (85.4)			
Previous Chemotherapy Status					
Chemotherapy Naïve	10 (20)	40 (80)	0.012	1	0.913
Received Chemotherapy	3 (18.8)	13 (81.3)			
Baseline Haemoglobin Value					
Hb<120	14 (14.3)	24 (85.7)	0.9	1	0.343
Hb>120	9 (23.7)	29 (76.3)			
Baseline Bilirubin Values					
Bilirubin<13	10 (29.4)	24 (70.6)	4.077	2	**0.043**
Bilirubin>13	2 (8)	23 (92)			
Baseline Albumin Values					
Alb<34	3 (12)	22 (88)	1.751	1	0.186
Alb>34	10 (25.6)	29 (74.4)			
Baseline Alkaline Phosphate Values					
ALP<180	10 (31.3)	22 (68.8)	4.474	1	**0.034**
ALP>180	3 (9.7)	28 (90.3)			
Neutrophil to Lymphocyte Ratio (NLR)					
NLR<4	10 (27)	27 (73)	5.147	1	**0.023**
NLR>4	1 (4.2)	23 (95.8)			
Number of Carbo/Gem Cycles Received					
Less than 4 cycles	4 (12.1)	29 (87.9)	2.824	1	0.093
More than 4 cycles	9 (29)	22 (71)			

S3.2 Bivariate analysis of Survival status at 6 months [Table S7]

Table S7. Bivariate analysis of Survival status at 6 months.

CRITERIA	Survival At 6 months		Pearson Chi Square		
	ALIVE	DECEASED	Value	df	χ^2 p-value
Sex					
Male	20 (51.3)	19 (48.7)	4.76	1	**0.029**
Female	21 (77.8)	6 (22.2)			
Age					
Age <72	22 (61.1)	14 (38.9)	0.034	1	0.853
Age>72	19 (63.3)	11 (36.7)			
Extent of Disease					
Locally Advanced	15 (65.2)	8 (34.8)	0.144	1	0.705
Metastatic	26 (60.5)	17 (39.5)			
Primary Site of Cancer					
Gall Bladder	4 (66.7)	2 (33.3)	5.046	4	0.283
Intra-Hepatic CCA	18 (51.4)	17 (48.6)			
Extra-Hepatic CCA	11 (73.3)	4 (29.7)			
Hilar	4 (66.7)	2 (33.3)			
Ampullary	4 (100)	0			
ECOG PS					
0	5 (100)	0	3.712	2	0.156
1	31 (60.8)	20 (39.2)			
2	5 (50)	5 (50)			
Lymph node involvement					
No LN involvement	26 (72.2)	10 (27.8)	3.434	1	0.064
LN involvement	15 (50)	15 (50)			
Previous Therapy Status					
No Previous Therapy	16 (66.7)	8 (33.3)	0.211	1	0.646
Received Previous Therapy	25 (61)	16 (39)			

Previous Chemotherapy Status					
Chemotherapy Naïve	32 (64)	18 (36)	0.309	1	0.578
Received Chemotherapy	9 (56.3)	7 (43.8)			
Baseline Ca19.9					
Ca 19.9 <127	22 (66.7)	11 (33.3)	0.58	1	0.447
Ca 19.9 >127	19 (57.6)	14 (42.4)			
Baseline Ca19.9					
Ca 19.9 <2000	34 (65.4)	18 (34.6)	1.109	1	0.292
Ca 19.9 >2000	7 (50)	7 (50)			
Baseline Haemoglobin Value					
Hb<120	16 (57.1)	12 (42.9)	0.512	1	0.474
Hb>120	25 (65.8)	13 (34.2)			
Baseline Bilirubin Values					
Bilirubin<13	28 (82.4)	6 (17.6)	13.237	1	**<0.01**
Bilirubin>13	9 (36)	16 (64)			
Baseline Albumin Values					
Alb<34	11 (44)	14 (56)	4.944	1	**0.026**
Alb>34	28 (71.8)	11 (28.2)			
Baseline Alkaline Phosphate Values					
ALP<180	24 (75)	8 (25)	5.857	1	**0.016**
ALP>180	14 (45.2)	17 (54.8)			
Neutrophil to Lymphocyte Ratio (NLR)					
NLR<4	26 (70.3)	11 (29.7)	3.643	1	0.056
NLR>4	11 (45.8)	13 (54.2)			
Number of Carbo/Gem Cycles Received					
Less than 4 cycles	11 (33.3)	22 (66.7)	21.809	1	**<0.001**
More than 4 cycles	28 (90.3)	3 (9.7)			
Dose Reduction Status					
Yes	13 (56.5)	10 (43.5)	0.759	1	0.384
No	27 (67.5)	13 (32.5)			

S3.3 Bivariate analysis of survival status at 12 months [Table S8]

Table S8. Bivariate analysis of survival status at 12 months.

CRITERIA	Survival At 12 months		Pearson Chi Square		
	ALIVE	DECEASED	Value	df	χ^2 p-value
Sex					
Male	11 (28.2)	28 (71.8)	0.574	1	0.449
Female	10 (37)	17 (63)			
Age					
Age <72	11 (30.6)	25 (69.4)	0.058	1	0.809
Age>72	10 (33.3)	20 (66.7)			
Extent of Disease					
Locally Advanced	9 (39.1)	14 (60.9)	0.87	1	0.351
Metastatic	12 (27.9)	31 (72.1)			
Primary Cancer Site					
Gall Bladder	2 (33.3)	4 (66.7)	7.229	4	0.124
Intra-Hepatic CCA	7 (20)	28 (80)			
Extra-Hepatic CCA	7 (46.7)	8 (53.3)			
Hilar	2 (33.3)	4 (66.7)			
Ampullary	3 (75)	1 (25)			
ECOG PS					
0	2 (40)	3 (60)	2.633	2	0.268
1	18 (35.3)	33 (64.7)			
2	1 (10)	9 (90)			
Lymph node involvement					
No LN involvement	16 (44.4)	20 (55.6)	5.82	1	0.016
LN involvement	5 (16.7)	25 (83.3)			
Previous Therapy Status					
No Previous Therapy	9 (37.5)	15 (62.5)	0.469	1	0.493
Received Previous Therapy	12 (29.3)	29 (70.7)			

Previous Chemotherapy Status					
Chemotherapy Naïve	16 (32)	34 (68)	0.003	1	0.955
Received Chemotherapy	5 (31.3)	11 (68.8)			
Baseline Ca19.9					
Ca 19.9 <127	11 (33.3)	22 (66.7)	0.7	1	0.792
Ca 19.9 >127	10 (30.3)	23 (69.7)			
Baseline Ca19.9					
Ca 19.9 <2000	17 (32.7)	35 (67.3)	0.086	1	0.769
Ca 19.9 >2000	4 (28.6)	10 (71.4)			
Baseline Haemoglobin Value					
Hb<120	8 (28.6)	20 (71.4)	0.236	1	0.627
Hb>120	13 (34.2)	25 (65.8)			
Baseline Bilirubin Values					
Bilirubin<13	15 (44.1)	19 (55.9)	7.01	1	0.08
Bilirubin>13	3 (12)	22 (88)			
Baseline Albumin Values					
Alb<34	5 (20)	20 (80)	3.055	1	0.08
Alb>34	16 (41)	23 (59)			
Baseline Alkaline Phosphate Values					
ALP<180	15 (46.9)	17 (53.1)	6.87	1	**0.009**
ALP>180	5 (16.1)	26 (83.9)			
Neutrophil to Lymphocyte Ratio (NLR)					
NLR<4	16 (43.2)	21 (56.8)	8.529	1	**0.003**
NLR>4	2 (8.3)	22 (91.7)			
Number of Carbo/Gem Cycles Received					
Less than 4 cycles	3 (9.1)	30 (90.9)	13.846	1	**<0.001**
More than 4 cycles	16 (51.6)	15 (48.4)			
Dose Reduction Status					
Yes	5 (21.7)	18 (78.3)	2.191	1	0.139
No	16 (40)	24 (60)			

S4. Toxicity Profiles [Table S9]

Table S9. Frequency table of toxicities from chemotherapy.

Adverse effects	Frequency n(%)
Neutropenia (Grade 1/2)	9 (13.7)
Neutropenia (Grade 3/4)	31 (31.82)
Neutropenia (Unknown Grade)	4 (6.06)
Thrombocytopenia (Grade 1/2)	26 (39.4)
Thrombocytopenia (Grade 3/4)	13 (19.7)
Thrombocytopenia (Unknown Grade)	3 (4.53)
Anaemia (Grade1/2)	14 (21.21)
Anaemia (Grade3/4)	16 (24.2)
Anaemia (Unknown Grade)	4 (6.06)
Nausea and Vomiting	21 (31.8)
Constipation and Diarrhoea	15 (22.72)
Peripheral Neuropathy	10 (15.2)
Others (SIADH, epistaxis, sore mouth, dyspepsia)	11 (16.7)
